# Supplementary material for: Clinical Routine TERT Promoter Mutational Screening of Follicular Thyroid Tumors of Uncertain Malignant Potential (FT-UMPs): A Useful Predictor of Metastatic Disease
Source: Cancers (Basel). 2019 Sep 26;11(10):1443. doi: 10.3390/cancers11101443 (PMC6826397; doi:10.3390/cancers11101443)
Supplement: Supplementary file 1 [file cancers-11-01443-s001.pdf]

Supplementary Materials

Clinical Routine *TERT* Promoter Mutational Screening of Follicular Thyroid Tumors of Uncertain Malignant Potential (FT-UMPs): A Useful Predictor of Metastatic Disease

Supplementary Table

Table 1. Previous reports regarding postoperative TERT promoter mutational status in unique series of FT-UMPs/AFTAs.

First Author	Journal	Year Published	No. of FT-UMPs / AFTAs Sequenced*	Number of Mutated Cases	Number of Mutated Cases with Relapse/Metastatic Disease
Wang N	*Cancer*	2014	18	3	0
Hysek M	*Virchows Arch*	2018	1	1	1
Juhlin CC	*Int J Surg Pathol*	2018	1	0	-
Duan H	*Diagn Pathol*	2019	32	0	-

FT-UMP; follicular tumor of uncertain malignant potential, AFTA; atypical follicular thyroid adenoma, -; not able to estimate. *FT-UMPs and AFTA consitute the recommended nomenclature in the WHO guidelines of 2017 and 2004 respectively
